# Armored long non-coding RNA MEG3 targeting EGFR based on recombinant MS2 bacteriophage virus-like particles against hepatocellular carcinoma

**DOI:** 10.18632/oncotarget.8115

**Published:** 2016-03-16

**Authors:** Le Chang, Guojing Wang, Tingting Jia, Lei Zhang, Yulong Li, Yanxi Han, Kuo Zhang, Guigao Lin, Rui Zhang, Jinming Li, Lunan Wang

**Affiliations:** ^1^National Center for Clinical Laboratories, Beijing Hospital, Beijing, People's Republic of China; ^2^Graduate School, Peking Union Medical College, Chinese Academy of Medical Sciences, Beijing, People's Republic of China; ^3^Department of Clinical Laboratory, Beijing Chaoyang Hospital, Capital Medical University, Beijing, People's Republic of China; ^4^Peking University Fifth School of Clinical Medicine, Beijing, People's Republic of China

**Keywords:** hepatocellular carcinoma, maternally expressed gene 3, MS2 virus-like particles, epidermal growth factor receptor, GE11

## Abstract

Hepatocellular carcinoma (HCC) is one of the most frequently diagnosed cancers worldwide. However, the treatment of patients with HCC is particularly challenging. Long non-coding RNA maternally expressed gene 3 (*MEG3*) has been identified as a potential suppressor of several types of tumors, but the delivery of long RNA remains problematic, limiting its applications. In the present study, we designed a novel delivery system based on MS2 virus-like particles (VLPs) crosslinked with GE11 polypeptide. This vector was found to be fast, effective and safe for the targeted delivery of lncRNA MEG3 RNA to the epidermal growth factor receptor (EGFR)-positive HCC cell lines without the activation of EGFR downstream pathways, and significantly attenuated both in vitro and in vivo tumor cell growth. Our study also revealed that the targeted delivery was mainly dependent on clathrin-mediated endocytosis and MEG3 RNA suppresses tumor growth mainly via increasing the expression of p53 and its downstream gene *GDF15*, but decreasing the expression of MDM2. Thus, this vector is promising as a novel delivery system and may facilitate a new approach to lncRNA based cancer therapy.

## INTRODUCTION

Hepatocellular carcinoma (HCC) is one of the most frequently diagnosed cancers worldwide, and is the second leading cause of cancer death globally [1]. However, treatment of patients with HCC is particularly challenging because of patient-specific, tumor-specific, and liver-specific variables [2]. In addition, current therapies for HCC produce poor long-term outcomes due to the development of drug resistance, the mechanisms of which are still unclear [3]. Therefore, we are interested in identifying novel drugs for effective treatment of HCC.

Long non-coding RNAs (lncRNAs) are broadly defined as endogenous cellular non-coding RNA molecules that are longer than 200 nucleotides; these lncRNAs perform multiple functions as signals, decoys, guides, and scaffolds [4, 5]. Recently, several lncRNAs have been shown to be associated with cancer [6–9]. The maternally expressed gene 3 (*MEG3*), the product of which functions as a non-coding RNA [10], is normally or highly expressed in the human pituitary, brain and meninges [11], lung [12], liver [13], bladder [14], and other human normal tissues [15–17]. However, its expression is lower or absent in some kinds of tumors and in several different human carcinoma cell lines, including HCC [18, 19]. Therefore, MEG3 may act as a novel tumor suppressor [20], and the association between MEG3 and cancer has received increasing attention. However, the only vector currently available for use with MEG3 is a plasmid vector containing a MEG3 cDNA fragment, and liposome-mediated transfection is the only method of delivery [12, 15–19]. In addition, deficiencies such as limited transduction efficiency, cytotoxicity, and integration-induced tumorigenesis, remain a concern that significantly limits the effect and applications of MEG3 therapy.

MS2 bacteriophage virus-like particles (VLPs) harboring specific RNA fragments (as long as 3,000 nt [21]) can easily be produced in *Escherichia coli* [22–26] or *Saccharomyces cerevisiae* [27, 28]. The VLP protein coat is resistant to various nucleases (DNase I or RNase A) [23, 27] and to changes in temperature and pH, within a certain range, both in vivo and in vitro [29, 30]. The VLP protein coat also contains many different amino acids, facilitating surface modification. All of these factors make it an ideal drug delivery vector. However, MS2 VLPs, as a biological macromolecule, cannot easily pass through the cell membrane, and are quickly removed from the cardiovascular system. Thus, a new method for mediating VLPs cell entry was required.

The dodecapeptide YHWYGYTPQNVI (GE11) was first identified as a ligand that binds to the epidermal growth factor receptor (EGFR) [31] but does not activate the receptor [32, 33]. It has been reported that GE11 could mediate passage of conjugated liposomes through the cell membrane [34–36]. EGFR is overexpressed on the surface of many carcinoma cells, including HCC [37, 38]. Therefore, in this study, we crosslinked the GE11 polypeptide to the surface of MS2 VLPs, thus rendering them target EGFR-positive cells. In the meanwhile the VLPs contained an lncRNA MEG3 RNA fragment, which acted as a tumor inhibitor. These targeted VLPs provide a new delivery strategy for therapy against EGFR-positive HCC.

## RESULTS

### Identification of VLPs and GE11-VLPs

**Figure 1:**
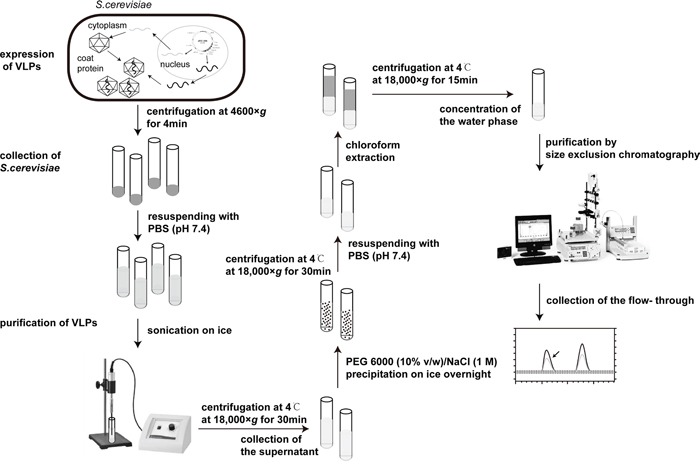
The flow chart of expression and purification of VLPs

**Figure 2:**
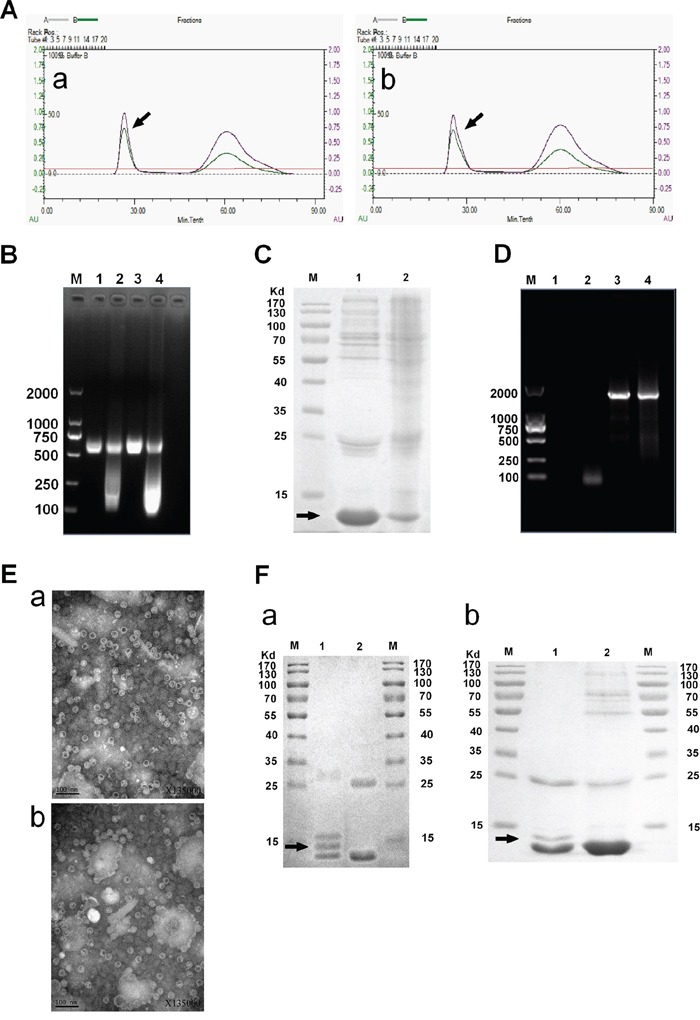
Identification of MS2 VLPs and GE11-VLPs **A.** Purification of VLPs. The peak of the target protein is marked by an arrow. **B.** Nuclease resistance assay of VLPs. The VLPs in Lane 1 and 3 were incubated with DNase I and RNase A, but those in Lanes 2 and 4 were not. Lanes 1 and 2, VLPs-MEG3; Lanes 3 and 4, VLPs-NC; Lane M, molecular mass marker. **C.** Verification of VLPs purity by SDS-PAGE. Lane 1, VLPs-MEG3; Lane 2, VLPs-NC; Lane M, molecular mass marker. The target protein is marked by an arrow. **D.** RT-PCR detection of lncRNA packaged by the MS2 capsid. Lane 1, negative control; Lane 2, VLPs-NC; Lane 3, VLPs-MEG3; Lanes 4, positive control; Lane M, molecular mass marker. **E.** Further verification of VLPs by TEM. a, VLPs-MEG3; b, VLPs-NC. **F.** Verification of GE11-VLPs by SDS-PAGE. a, Lane 1, GE11-VLPs-MEG3; Lane 2, VLPs-MEG3; M, molecular mass marker. b, Lane 1, GE11-VLPs-NC; Lane 2, VLPs-NC; M, molecular mass marker. The protein band after crosslinking is marked by an arrow.

To facilitate packaging of the MEG3 cDNA sequence in the MS2 coat protein, we inserted two mutated pac site sequences at the 5′- and 3′-termini of a MEG3 cDNA, and then expressed the MS2 VLPs in *S. cerevisiae*. After a series of purification steps (Figure 1), we obtained 5–6 mg of purified MS2 VLPs, prepared from 1 L of culture medium (Figure 2A). We further identified the MS2 VLPs using three approaches. First, the results of agarose gel electrophoresis with GelRed staining showed that after MS2 VLPs were incubated with DNase I and RNase A, a band of approximately 500–750 bp could still be observed (lanes 1–4, Figure 2B), indicating that the armored RNAs were nuclease-resistant. Secondly, sodium dodecyl sulfate polyacrylamide gel electrophoresis (SDS-PAGE) results revealed that the molecular weight of the MS2 coat protein was approximately 10–14 kDa (Figure 2C), and transmission electron microscope (TEM) results indicated that the VLPs had a diameter of approximately 26–30 nm (Figure 2E). Finally, the RNAs encapsulated in the VLPs were extracted and analyzed by RT-PCR. The results indicated that the VLPs-MEG3 contained an approximate 1600-bp MEG3 RNA sequence and that the VLPs-NC did not contain the specific RNA fragment (Figure 2D).

Then, we crosslinked MS2 VLPs-MEG3 and VLPs-NC with the polypeptide GE11-Cys using the Sulfo-SMCC crosslinker, and verified the effect by SDS-PAGE (Figure 2F). We identified the crosslinking band with a bit larger molecular weight than VLP bands, indicating that crosslinking was successful.

### Decreased expression of lncRNA MEG3 in HCC cells

**Figure 3:**
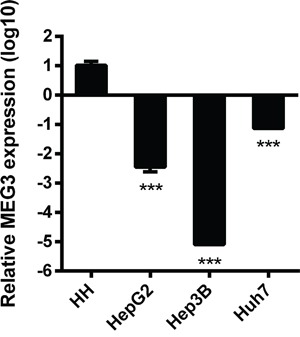
The base relative expression level of MEG3 Relative to Human Hepatocytes (HH), the expression level of MEG3 in HepG2, Hep3B and Huh7 varies from 10^−2^~10^−5^ fold (n=3). ***, p<0.001. Data are represented as mean ± SD.

To investigate the levels of lncRNA MEG3 expressed in normal hepatocytes and HCC cells, we isolated total RNA from human normal hepatocytes, and three HCC cell lines (HepG2, Hep3B, and Huh7) and performed RT-qPCR to quantify MEG3 and GAPDH mRNA. The expression of MEG3 mRNA was remarkably reduced in all three HCC cell lines, especially in Hep3B, compared with human normal hepatocytes (Figure 3).

### GE11-VLPs-MEG3 realize effective delivery of MEG3 RNA in vitro, dependent on EGFR endocytosis

**Figure 4:**
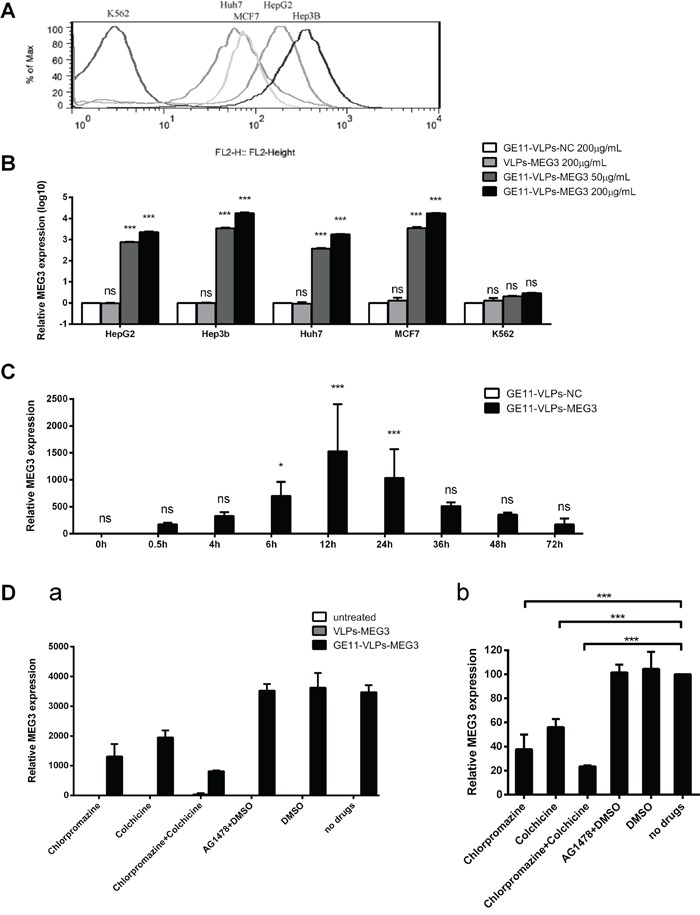
The effective delivery of MEG3 RNA via GE11-VLPs-MEG3 in different cell lines **A.** EGFR level on the surface of HepG2, Hep3B, Huh7, MCF-7 and K562 cell lines. **B.** The relative expression level of MEG3 in different concentration of VLPs treated different cell lines. GE11-VLPs-NC (200μg/mL), VLPs-MEG3 (200μg/mL), low concentration of GE11-VLPs-MEG3 (50μg/mL) and high concentration of GE11-VLPs-MEG3 (200μg/mL) treated HepG2, Hep3B, Huh7, MCF-7 and K562 cell lines. The relative expression level of MEG3 was analyzed by RT-qPCR (n=3). ***, p<0.001; ns, not significant. **C.** The relative expression level of MEG3 at different time after the treatment of VLPs in HepG2 cell lines. 0 h, 0.5 h, 4 h, 6 h, 12 h, 16 h, 24 h, 36 h, 48 h, 72 h after treatment of GE11-VLPs-NC and GE11-VLPs-MEG3 were analyzed (n=3). The relative expression level of MEG3 was analyzed by RT-qPCR. *, p<0.05; ***, p<0.001; ns, not significant. **D.** The relative expression level of MEG3 in HepG2 cell lines treated by different inhibitors. (a) The relative expression level of MEG3 treated by different VLPs and different inhibitors (n=3). (b) The relative expression level of MEG3 treated by GE11-VLPs-MEG3 and different inhibitors. The results were calculated relative to the no drug group (n=3). ***, p<0.001. All the data above are represented as mean ± SD.

First, we used flow cytometry analysis to evaluate the EGFR levels on the surface of different cell lines, including the EGFR-positive cell lines Hep3B, HepG2, Huh7, and MCF-7, and the EGFR-negative cell line K562 (Figure 4A). Then, we treated these cell lines with different concentrations of GE11-VLPs-MEG3, extracted total RNA from the cells after incubation for 24 h, and performed RT-qPCR to analyze MEG3 and GAPDH mRNA levels. The results showed high levels of MEG3 mRNA in Hep3B, HepG2, Huh7, and MCF-7 cells treated with GE11-VLPs-MEG3; the fold change ranged from 10^3^ to 10^5^, and was dose-dependent (Figure 4B). However, almost no increase was observed in K562 cells and cells treated with VLPs-MEG3 or GE11-VLPs-NC, indicating that entry of VLPs was mediated by the GE11 peptide and was dependent on GE11-EGFR interactions.

Analysis of different time points showed that after GE11-VLPs-MEG3 were incubated with HepG2 cells, VLPs began to enter cells within 30 min, the concentration reached a peak at approximately 12 h, and concentrations of VLPs remained high until 72 h (Figure 4C). Therefore, GE11-mediated entry of VLPs was also time-dependent.

To further explore the correlation between GE11-mediated entry of VLPs into cells and the EGFR signaling pathway, we performed inhibitor assays using the EGFR endocytosis inhibitors chlorpromazine and colchicine, as well as Tyrphostin AG 1478, a drug that specifically blocks phosphorylation of EGFR, in the solvent DMSO. Chlorpromazine was used to inhibit clathrin-mediated endocytosis via the dissociation of the clathrin lattice [39, 40]. After treatment with chlorpromazine, the MEG3 mRNA level decreased to 37.67% ± 12.31%, compared with the untreated group (Figure 4D), indicating that clathrin-mediated endocytosis was involved in cellular entry of GE11-VLPs. After treatment with colchicine, a specific inhibitor of micropinocytosis [41, 42], and the MEG3 mRNA level decreased to 56.08% ± 6.74% of controls (Figure 4D). Following treatment with both chlorpromazine and colchicine, cellular entry of GE11-VLPs was strongly inhibited, and was reduced to approximately 20%. Following treatment with AG1478 dissolved in DMSO and only DMSO, the uptake efficiency of GE11-VLPs and untargeted VLPs was not affected (Figure 4D), indicating uptake without EGFR activation. These results suggested that GE11-mediated entry of VLPs was mainly via clathrin-mediated endocytosis and micropinocytosis and cellular entry of GE11-VLPs did not activate the EGFR signaling pathway.

### GE11-VLPs-MEG3 could significantly inhibit proliferation, invasion, and colony formation of tumor cells

**Figure 5:**
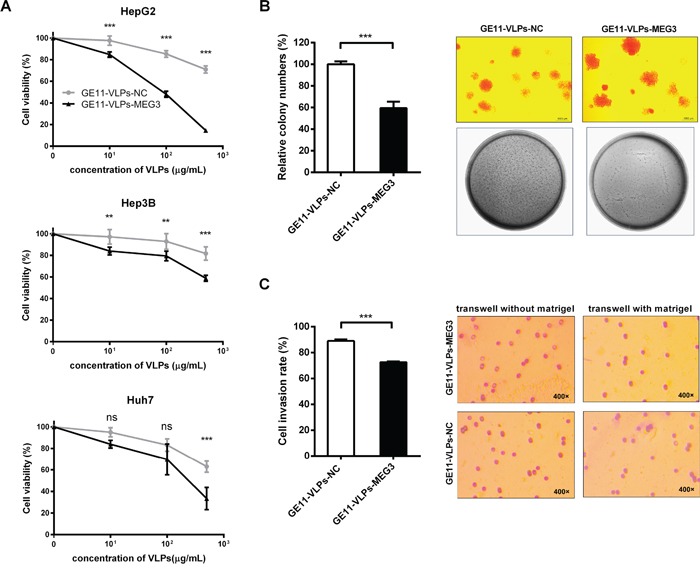
MEG3 suppresses cell proliferation, colony formation and cell invasion **A.** CCK-8 assay was performed to determine the proliferation of three types of hepatocellular carcinoma cell lines, HepG2, Hep3B and Huh7 treated with GE11-VLPs-NC and GE11-VLPs-MEG3 (n=3). **, p<0.01; ***, p<0.001; ns, not significant. **B.** Colony-forming growth assays were performed to determine the proliferation of HepG2 cells (n=3). The colonies were counted and captured. ***, p<0.001. **C.** Transwell cell invasion assays were performed to determine the invasion of HepG2 cells (n=3). The cells migrated through the membrane or matrigel were counted and captured. ***, p<0.001. All the data above are represented as mean ± SD.

To determine the biological role of MS2-encapsulated MEG3 in HCC cells, we investigated the effects of overexpression of MEG3 on cell proliferation. CCK-8 assays revealed that cell growth was significantly impaired in all the three HCC cell lines, especially in HepG2 cells (Figure 5A). Therefore, we chose the HepG2 cell line, the cell type that showed the most significant inhibition effect, for use in subsequent experiments.

To further examine the influence of MEG3 on clonogenic survival of tumor cells, we performed a colony formation assay on soft agar. HepG2 cells treated with GE11-VLPs-MEG3 formed 59.44% ± 6.03% colonies compared with control (Figure 5B), indicating the capability of colony formation of HepG2 cells was significantly inhibited.

Cell invasion is a significant aspect of cancer progression, and involves the migration of tumor cells into contiguous tissues and the dissolution of extracellular matrix proteins. Similarly, the results of cell invasion assays showed that fewer HepG2 cells treated with GE11-VLPs-MEG3 passed through the Matrigel in the Transwell assay, than that of the cells in the control group (72.62% ± 0.59% vs 89.00% ± 1.26%) (Figure 5C).

### GE11-VLPs-MEG3 promote cell apoptosis

**Figure 6:**
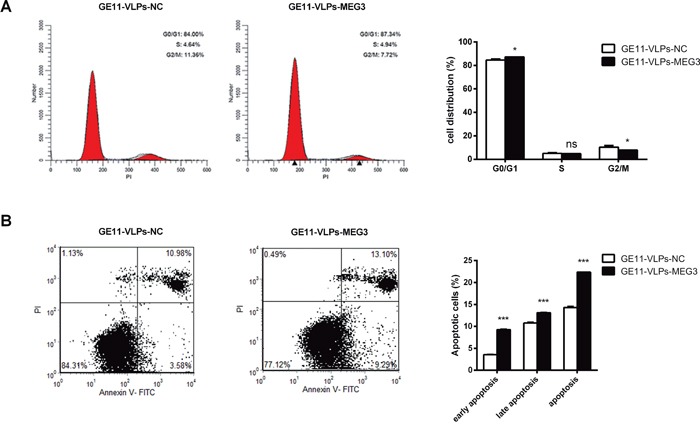
MEG3 arrests cell cycle and promotes cell apoptosis **A.** The cell cycle of HepG2 cells were detected by flow cytometry and the bar chart represented the percentage of cells in G0/G1, S, or G2/M phase (n=3). *, p<0.05; ns, not significant. **B.** The apoptotic rates of HepG2 cells were detected by flow cytometry (n=3). ***, p<0.001. All the data above are represented as mean ± SD.

To investigate whether MEG3 affected proliferation of HepG2 cells via cell cycle regulation, cell cycle progression was analyzed with propidium iodide (PI) staining and flow cytometry. The results revealed that HepG2 cells treated with GE11-VLPs-MEG3 showed cell cycle arrest at the G1/G0 phase and a slightly decreased G2/S phase (Figure 6A).

Cell apoptosis also contributes to inhibition of cell growth. Therefore, we performed a cell apoptosis analysis using double staining with Annexin V-FITC and PI followed by analysis of flow cytometry. Both early apoptosis and late apoptosis can be seen in Figure 6B, and the total apoptosis rate of the GE11-VLPs-MEG3-treated group was higher than that of the control groups.

### MEG3 functions related to p53

**Figure 7:**
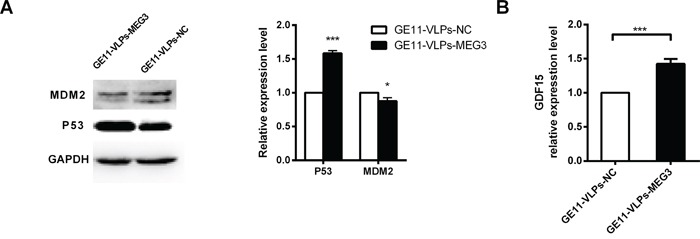
MEG3 increases p53 activation **A.** Western blot analysis of p53, MDM2 after treatment of GE11-VLPs-MEG3 and GE11-VLPs-NC. GAPDH protein was used as an internal control. *, p<0.05; ***, p<0.001. **B.** The relative expression of GDF15 were analyzed by RT-qPCR (n=3). ***, p<0.001. All the data above are represented as mean ± SD.

p53 plays a role in several processes involved in tumor proliferation, apoptosis, and metastasis. In this study, we found that HepG2 cells treated with GE11-VLPs-MEG3 for 48 h expressed higher levels of P53 protein than negative controls (Figure 7A). p53 is primarily regulated by the E3 ubiquitin ligase MDM2 (murine double minute 2), which binds p53 at its transactivation domain, blocking p53-mediated transcriptional regulation, simultaneously promoting its polyubiquitination and proteasome-dependent degradation [43]. The decreased expression level of MDM2 verified the function of MEG3 in the p53-related pathway (Figure 7A). In addition, we found that mRNA of growth differentiation factor 15 (GDF15), a downstream gene of p53 that plays an important role in apoptotic pathways, was significantly higher in the GE11-VLPs-MEG3 group than in controls (Figure 7B), indicating that MEG3 may operate via the MDM2-p53-GDF15 pathway.

### GE11-VLPs-MEG3 inhibits tumor growth in vivo

**Figure 8:**
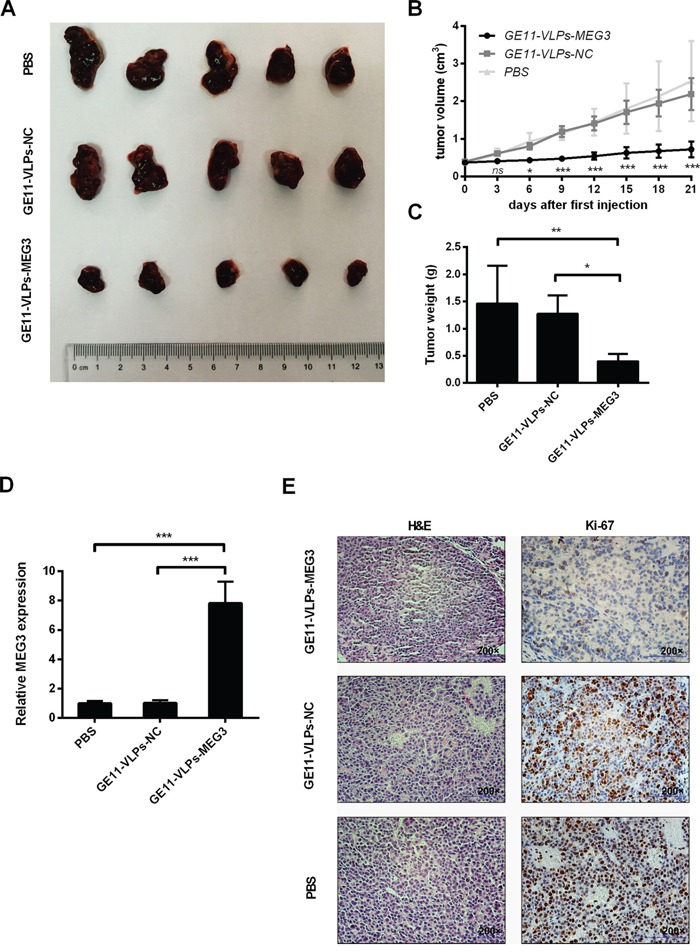
Effects of MEG3 on tumor growth in vivo **A.** Tumors were captured after three weeks’ treatment of GE11-VLPs-MEG3, GE11-VLPs-NC and PBS. **B.** The tumor volume was calculated every three days after the first treatment of VLPs (n=5). *, p<0.05; ***, p<0.001; ns, not significant. **C.** The tumors were weighed after taking out (n=5). *, p<0.05; **, p<0.01. **D.** RT-qPCR analysis of MEG3 expression in tumor tissues treated with GE11-VLPs-MEG3, GE11-VLPs-NC and PBS (n=3). ***, p<0.001. **E.** Tumors treated with GE11-VLPs-MEG3 showed lower Ki-67 protein levels than tumors treated with GE11-VLPs-NC or PBS. Left: H&E staining; Right: Ki-67 immunostaining. All the data above are represented as mean ± SD.

To investigate GE11-VLPs-MEG3 inhibition of tumor growth in vivo, we constructed a nude mouse model of HCC. Approximately 30 days after inoculation of HepG2 cells, all BALB/c nude mice developed tumors in the armpit area that were visible to the naked eye. We chose the mice almost with the similar size (<0.5 cm^3^) of tumor volumes, randomly divided them into three groups and treated them with different VLPs. We recorded tumor size every three days after therapy. Tumor growth in the GE11-VLPs-MEG3 group was significantly slower than that in the GE11-VLPs-NC and PBS groups (Figure 8A and 8B), indicating that GE11-VLPs-MEG3 could significantly inhibit tumor growth in vivo. Similarly, the final tumor mass in the GE11-VLPs-MEG3 group was much less than in the two control groups (Figure 8C). Tumor tissues were analyzed by RT-qPCR for MEG3 mRNA. Levels of MEG3 mRNA in tumor tissues from the GE11-VLPs-MEG3 group were higher than in the control groups (Figure 8D).

Immunohistochemistry and H&E staining were performed. Ki-67 is a nuclear protein that is associated with and may be necessary for cell proliferation. Ki-67 staining of the GE11-VLPs-MEG3 group was significantly lower than the controls (Figure 8E). These results indicated that GE11-VLPs-MEG3 could enter tumor cells and inhibit cell proliferation in vivo.

## DISCUSSION

**Figure 9:**
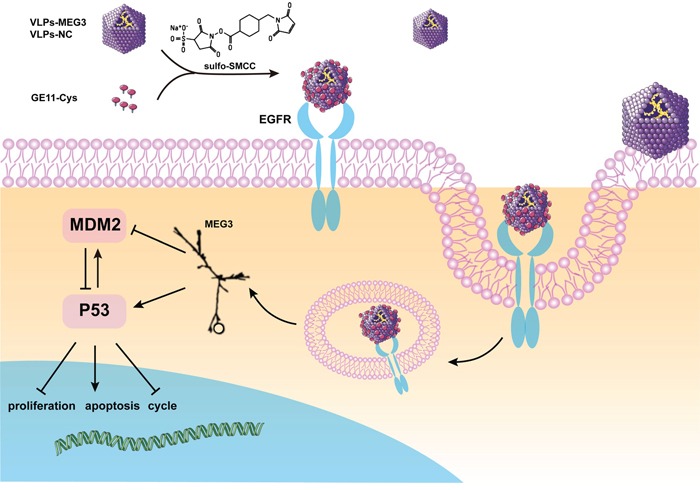
Schematic diagram of the construction of GE11-VLPs-MEG3 and its function in cells *The figure of MEG3 is cited from Zhou et al. [48].

In this study, we packaged lncRNA MEG3 RNA into MS2 VLPs surface-crosslinked with the GE11 polypeptide. These GE11-VLPs-MEG3 could pass through the cell membrane in an EGFR-dependent manner and acted as tumor suppressors against HCC in vivo and in vitro, via a p53-related signaling pathway (Figure 9).

All presently available studies report that insertion of MEG3 cDNA into plasmids coupled with liposome-mediated cell transfection is the only method to realize tumor inhibition. However, as a vector, plasmids have many disadvantages. Firstly and most importantly, plasmids are rapidly cleared from the cardiovascular system, cannot be targeted to particular tissues or cells, and cannot pass through the cell membrane smoothly. Secondly, liposomes can cause some degree of cell damage, although available reagents are improving. Finally, integration-induced tumorigenesis, although the likelihood is very low, can occur when plasmids are incorporated into the cell. In addition, due to its instability, RNA delivery in vitro or in vivo often requires complicated procedures and is difficult to target to cells. Thus, an effective MEG3 RNA treatment has yet to be developed.

The main finding of this study is that MS2 VLPs can be used as a vector to realize delivery of lncRNA MEG3 RNA. The MS2 bacteriophage is an icosahedral, positive-sense single-stranded RNA virus, composed of 90 coat protein dimers. We obtained large quantities of MS2 VLPs simply by inserting the coat protein cDNA sequence into the pESC-URA plasmid and expressing them in *S. cerevisiae*. VLPs themselves are stable at various temperatures and pH, and can resist degradation by ribonucleases, thus protecting the encapsulated RNA from degradation [21, 29, 30]. After crosslinking with the GE11 polypeptide, VLPs could target EGFR-positive cells, especially tumor cells, which greatly reduce the possibility of side effects. In addition, although the MS2 bacteriophage is a type of bacterial virus, MS2 VLPs contain only coat protein and no genomic RNA. Therefore, there is no possibility of integration-induced tumorigenesis. Liposomes have recently been widely used as a vector for DNA or RNA delivery, but the process required for their preparation is more complicated than that for VLPs, and their diameter is >100 nm [44], approximately 4-fold that of MS2 VLPs, making it more difficult for them to enter cells. In view of these advantages, this study adopted an MS2 VLP-based delivery approach, in which the MEG3 RNA fragment is encapsulated by the MS2 bacteriophage coat protein, to which the GE11 polypeptides are chemically crosslinked.

However, there are also some drawbacks of VLPs as a vector for lncRNA therapy, which needs to be further improved in the future. In vivo use of MS2 VLPs, as protein biomacromolecules, could provoke an immune response unavoidably, and they would be cleared away by macrophages or other immune cells, resulting in decreased concentration of the drug and reduced effectiveness. In this study, after 3 weeks’ therapy, we could detect higher titers of anti-MS2 coat protein IgG antibody in the serum of mice treated with GE11-VLPs-MEG3 and GE11-VLPs-NC compared with that with PBS. In addition, a relatively lower level of MEG3 mRNA was measured in tumor tissues treated with GE11-VLPs-MEG3 than in cells in vitro culture. Although the immune response to VLPs weakened the drug effect and drug concentration, tumor growth was still significantly inhibited, and no obvious serious side effects were observed in mice. Another drawback to this method is that the length of RNAs that can be encapsulated in MS2 VLPs is limited. To date, the maximum length of an RNA that can be packaged in VLPs is 3034 nt [21]. Since the length of lncRNA varies from 200 nt to 100,000 nt, some longer lncRNAs could not be packaged into VLPs in vivo, thus limiting the use of MS2 VLPs for delivery of lncRNAs, although MS2 VLPs have successfully been used for quality control of viral nucleic acids [21, 23, 24, 26, 45, 46] and microRNA therapy [22, 28, 47].

In this study, a large number of GE11-VLPs-MEG3 entered EGFR-positive HepG2, Hep3B, Huh7, and MCF-7 cells, and only a two- or three-fold increase was observed in EGFR-negative K562 cells compared with controls. In all cell lines treated with VLPs-MEG3 without GE11 crosslinking, the level of MEG3 RNA showed almost no change compared with controls. These facts indicated that entry of GE11-VLPs into cells might be related to EGFR. The small increase in MEG3 observed in K562 cells might have been caused by the presence of a few VLPs on the cell surface, or by another unknown mechanism independent of EGFR. Further exploration showed that GE11-mediated cell entry could be inhibited by chlorpromazine and colchicine, but not by Tyrphostin AG 1478 and its solvent DMSO. Cheng et al. [44] showed that GE11-mediated entry of the cells could be inhibited by chlorpromazine and colchicine, but not by filipin. However, colchicine could also inhibit PEG-liposomes from entering the cells, indicating that chlorpromazine is a specific inhibitor for GE11-mediated entry of cells and that GE11 exerts its function via clathrin-mediated endocytosis. In addition, Mickler et al. [32] found that AG 1478, a specific inhibitor of EGFR phosphorylation, could inhibit EGF from entering cells but could not inhibit GE11, indicating that GE11-mediated cancer therapy does not cause side effects via mitogenic signaling.

The tumor suppressor p53 plays a central role in tumor suppression and tumorigenesis. It is reported that MEG3 can inhibit cell proliferation and promote cell apoptosis through a p53-related pathway [48]. Our results also confirmed that as the intracellular MEG3 levels increased, p53 expression in HepG2 cells significantly increased compared with controls; however, MDM2 decreased compared with the GE11-VLPs-NC group. p53 can be degraded by MDM2 via polyubiquitination and proteasome-dependent pathways, however, p53 can also enhance MDM2 transcription through p53-specific response elements in the promoter region of MDM2, thus forming an autoregulatory feedback loop to control the balance of p53 and MDM2 in vivo [43, 49]. We think that increased levels of intracellular MEG3 may inhibit MDM2 and promote p53, thus upset the balance. However, there are no evidence showing whether MEG3 RNA direct functions on p53 and/or MDM2 or how it functions. There have been two possible speculations proposed by Zhou et al. [48]. One possibility is that MEG3 may function as a transcriptional co-activator (it has been shown ncRNAs can act as co-activators [50]) to stimulate expression of proteins that modify p53 and/or MDM2. The other is that MEG3 RNA may form complexes with MDM2 and/or p53 to block MDM2-mediated p53 degradation. In addition, expression levels of genes downstream of p53 were also altered. For example, GDF15, which belongs to the transforming growth factor beta superfamily and which plays a role in regulating inflammatory and apoptotic pathways has been shown to inhibit proliferation of several cancer cell lines, including the breast cancer line MDA-MB-468 and MCF7 [51], and the colon cancer line HCT116 [52]. Therefore, the MDM2-p53-GDF15 pathway may be important in the tumor-suppressor activity of MEG3.

In conclusion, the MS2 VLP-based lncRNA therapy strategy discussed here has many advantages, making it a promising vector for delivery of MEG3 on cancer therapy. GE11-mediated entry of VLPs into cells is a fast, effective, and safe way to realize targeted delivery to EGFR-positive cells that is mainly dependent on clathrin-mediated endocytosis. MEG3 RNA packaged in MS2 VLPs can functions as a tumor suppressor and significantly inhibits cell proliferation, cell invasion, and colony formation, arrests the cell cycle, and promotes apoptosis of HCC cells in vitro and in vivo, perhaps via the MDM2-p53-GDF15 pathway. Although there were limitations, MS2 VLPs-based lncRNA therapy shows promise for treatment of cancer, but will require much further research.

## MATERIALS AND METHODS

### Cell culture

Human normal hepatocytes were purchased from Creative Bioarray (Shirley, NY). The HepG2, Huh7, and MCF-7 cell lines were cultured in Dulbecco's modified Eagle medium (DMEM) (Gibco, Grand Island, NY) supplemented with 10% fetal bovine serum (FBS) (Gibco, Grand Island, NY). The Hep3B cell line was cultured in minimum essential medium (MEM) (Gibco, Grand Island, NY) supplemented with 15% FBS. These four cell lines were obtained from ATCC (Manassas, VA). The K562 cell line was purchased from China Infrastructure of Cell Line Resource and cultured in RPMI 1640 (Gibco, Grand Island, NY) supplemented with 10% FBS. All cell lines were cultured in medium supplemented with 100 U/mL penicillin and 100 μg/mL streptomycin (Gibco, Grand Island, NY) in a humidified incubator at 37°C with 5% CO_2_.

### Construction of plasmids

The cDNA sequence of the MS2 bacteriophage coat protein was cloned from pESC-URA-MS2 (archived in our laboratory) using the primers CP-F and CP-R, then inserted into the *Xho* I and *Bam* HI sites of the pESC-URA vector. This new plasmid was named pESC-MS2. A total of 16 isoforms of MEG3 RNA (transcript variants 1–16) have been found; 12 have been identified and named MEG3, and MEG3a to MEG3k. In this study, we chose the predominantly expressed isoform, MEG3, harboring exons 1–4 and 8–10 (GenBank accession number: NR_002766.2) [53, 54] for packaging into MS2 VLPs.

The cDNA sequence of MEG3 was cloned from pCDNA3.0-MEG3 (kindly provided by Professor Xiaofei Zheng of the Beijing Institute of Radiation Medicine) using the PCR primers MEG3-F and MEG3-R, which both contain a mutated pac site sequence to aid MS2 coat protein encapsulation of the specific RNA fragments 39 then inserted into *Spe* I and *Pac* I of pESC-MS2. This new plasmid was named pESC-MS2-MEG3. Correct construction of all plasmids was confirmed by sequencing. All restriction enzymes and T4 ligase were obtained from Thermo Fisher Scientific (Grand Island, NY). All primer sequences are listed in Table 1.

**Table 1:** Primer sequences

primer	sequence
CP-F*	5’-CGCGGATCCGCCACCATGGCTTCTAAC-3’
CP-R*	5’-CCGCTCGAGATGGCCGGCGTCTATTA-3’
MEG3-F*	5’-CCGACTAGT**ACATGAGGAT*C*ACCCATGT**AGCCCCTAGCGCAGAC-3’
MEG3-R*	5’-GCCTTAATTAA**ACATGGGTGATCCTCATGT**TTTTGTTAAGACAGGAAACACATTTATTGAG-3’
MEG3-q-F	5’-ATCATCCGTCCACCTCCTTGTCTTC-3’
MEG3-q-R	5’-GTATGAGCATAGCAAAGGTCAGGGC-3’
GDF15-F	5’-TGCCCGCCAGCTACAATC-3’
GDF15-R	5’-TCTTTGGCTAACAAGTCATCATAGGT-3’
GADPH-F	5’-AATGCCTCCTGCACCACCAAC-3’
GADPH-R:	5’-AAGGCCATGCCAGTGAGCTTC-3’.

Note: *Sequences under the line are restriction enzyme recognition site. Sequences in bold are mutated pac site sequences.

### Preparation and identification of MS2 VLPs

The plasmids pESC-MS2 and pESC-MS2-MEG3 were transferred into the YPH499 yeast strain (Agilent Technologies, Santa Clara, CA) by the LiAC/ssDNA/PEG method [27]. The expression of VLPs was performed according to the instructions for the pESC-URA yeast epitope tagging vector (Agilent Technologies, Santa Clara, CA). Yeast cells from 1L culture were harvested by centrifugation, resuspended in a final volume of 30 mL PBS (pH 7.4) and disrupted by sonication (Branson Sonic Sonifier 350, Emerson Electric Company Danbury, CT) on ice for 40 min (on for 5 sec, off for 8 sec, power 50%). The homogenate was centrifuged at 4°C for 30 min at 18,000× *g*, then the supernatant was collected and the VLPs were recovered from the supernatant by PEG 6000 (10% v/w)/NaCl (1 M) precipitation on ice overnight. After centrifuged at 4°C for 30 min at 18,000× *g*, the precipitate was resuspended in 20 mL PBS (pH 7.4) and then 20 mL chloroform was added and mixed well. The mixture was centrifuged at 4°C for 15 min at 18,000× *g*. The supernatant was collected, and then concentrated in a dialysis bag by PEG20000. Finally, the concentrated solution was purified by Sephacryl A-1.5 m gel (BioLogic DuoFlow chromatography system, Bio-Rad, Hercules, CA) based on the method of size exclusion chromatography.

The products were first verified by 1% agarose gel electrophoresis with GelRed (Biotium, Hayward, CA) staining before and after incubated with 1μl DNase I (10 U/mL) and 1μl RNase A (10 mg/mL) (Sigma, St Louis, MO) at 37°C for 3 h. Secondly, the products were analyzed by SDS–PAGE on 12% gels and were also observed by TEM at 75 kV and 200,000× screen magnification. Finally, RNA was extracted from the two types of purified VLPs (VLPs-NC and VLPs-MEG3), using a QIAamp Viral RNA Mini Kit (QIAGEN, Hilden, Germany). RT-PCR analysis was performed using the PCR primers MEG3-F and MEG3-R with the QIAGEN OneStep RT-PCR Kit (QIAGEN, Hilden, Germany). The amplified PCR products were analyzed by 1% agarose gel electrophoresis with GelRed staining.

### Preparation and identification of GE11-VLPs

Detection of protein concentrations of VLPs-NC and VLPs-MEG3 was performed using BCA assays (Beyotime Biotechnology, Beijing, China), according to the manufacturer's instructions. A cysteine residue was added to the N-terminus of the GE11 polypeptides for crosslinking to the surface of MS2 VLPs, which were synthesized by Chinese Peptide Company (Hangzhou, China). The crosslinker reagent sulfosuccinimidyl 4-(N-maleimidomethyl) cyclohexane-1-carboxylate (Sulfo-SMCC) was obtained from Thermo Fisher Scientific (Rockford, IL). The VLPs-NC and VLPs-MEG3 were crosslinked with the GE11-Cys polypeptide according to the manufacturer's instructions. SDS-PAGE on 12% gels was performed to verify crosslinking.

### Flow cytometry analysis of cell surface EGFR

HepG2, Huh7, Hep3B, MCF-7, and K562 cells were harvested, washed with PBS (pH 7.4) twice, and counted. Cells (1 × 10^6^) were suspended in 100 μl PBS (pH 7.4), then incubated with 10μl PE-anti-EGFR antibody (BD, Franklin Lakes, NJ) or 10μl isotype control antibody (BD, Franklin Lakes, NJ), respectively at 4°C for 30 min and washed five times with PBS (pH 7.4) followed by analysis using flow cytometry (BD FACSCalibur, Heidelberg, Germany).

### RNA extraction and qRT-PCR analysis

Total RNA was isolated with TRIzol reagent (Invitrogen, Carlsbad, CA, USA) according to the manufacturer's protocol. Total RNA (500 ng) was reverse transcribed in a final volume of 10 μl using random primers and oligo dT primers under standard conditions using the PrimeScript RT reagent Kit (TaKaRa, Dalian, China) and then qPCR was performed with SYBR Premix Ex Taq II (TaKaRa, Dalian, China) according to the manufacturer's instructions.

The relative levels of MEG3 and GDF15 were determined by qPCR using gene-specific primers. GAPDH was used as an internal control, as its expression shows little variation in different cell lines and cancer specimens. The RT reaction was carried out under the following conditions: 37°C for 15 min; 85°C for 5 sec; with a hold at 4°C using the random primers. After the RT reaction, 2 μl of the complementary DNA was used for subsequent qPCR reactions. The qPCR reaction was conducted at 95°C for 5 sec and followed by 40 cycles of 95°C for 5 sec and 60°C for 34 sec in an ABI 7500 real-time PCR system (Applied Biosystems, Foster City, CA, USA) using the qPCR primers MEG3-q-F, MEG3-q-R, GDF-F, GDF-R, GAPDH-F, and GAPDH-R. The qPCR results were analyzed relative to the Ct (threshold cycle) value, and converted to fold-change values according to the rules of 2^−ΔΔCt^. Sequences of all primers are listed in Table 1.

### In vitro drug delivery and inhibition studies

The HCC cell lines HepG2, Huh7, and Hep3B, the breast cancer cell line MCF-7, and EGFR-negative K562 cells were seeded at 2 × 10^5^ cells on 24-pore plates and incubated in culture medium overnight. After the cells reached approximately 80% confluence, GE11-VLPs-MEG3, VLPs-MEG3, and GE11-VLPs-NC were diluted in culture medium at two different concentrations (final concentrations of 50 μg/mL and 200 μg/mL) and added to the culture plates. After 24 h incubation at 37°C, the cells were washed six times with 1mL PBS (pH 7.4) to remove unbound VLPs and the cells were prepared for RT-qPCR analysis.

For the different time point analysis, after the cells reached approximately 80% confluence, GE11-VLPs-MEG3, VLPs-MEG3, and GE11-VLPs-NC were diluted in culture medium (final concentrations were 50 μg/mL) and added to the plates. After 30 min, 4 h, 6 h, 12 h, 24 h, 36 h, 48 h, and 72 h incubation at 37°C, the cells were washed six times with 1mL PBS (pH 7.4) to remove unbound VLPs and the cells were prepared for RT-qPCR analysis.

For the delivery inhibition study, after the cells reached approximately 80% confluence, different EGFR-related inhibitors were added to the wells. These inhibitors included the EGFR endocytosis inhibitors chlorpromazine (10 μg/mL) (Sigma, St Louis, MO) and colchicine (40 μg/mL) (Solarbio, Beijing, China), the EGFR tyrosine kinase inhibitor Tyrphostin AG 1478 (10 μM) (Sigma, St Louis, MO), and its solvent DMSO (Amresco, Solon, OH). After 3 h incubation at 37°C, GE11-VLPs-MEG3 and GE11-VLPs-NC were added to the plates at a final concentration of 50 μg/mL and the plates were incubated for 12 h. Cells were washed six times with 1mL PBS (pH 7.4) to remove unbound VLPs and the cells were prepared for RT-qPCR analysis. All experiments were performed in triplicate.

### Cell proliferation assays

Cell proliferation was monitored using a Cell Counting Kit-8 assay (CCK-8) (Dojindo, Kumamoto, Japan). The HCC cell lines HepG2, Huh7, and Hep3B were seeded at 1 × 10^3^ cells on 96-pore plates and incubated in culture medium overnight. After the cells reached approximately 50%, 100μl of different concentrations of GE11-VLPs-MEG3 and GE11-VLPs-NC (10 μg/mL to 500 μg/mL) were administered to the HepG2, Hep3B, and Huh7 cell lines in 96-well plates for 72 h. CCK-8 reagent (10 μl) was added to every well and incubation continued for 2 h, followed by measurement of absorbance at 450 nm on a Thermo Scientific Multiskan FC (Vantaa, Finland). All experiments were performed in triplicate.

### Colony formation assay

HepG2 cells were seeded in 6-well plates (5 × 10^5^ cells per well) in DMEM medium with 10% FBS and treated for 48 h with 20 μg/mL of GE11-VLPs-MEG3 and GE11-VLPs-NC. Then cells were digested by trypsin with 0.05% EDTA (Thermo Fisher Scientific, Waltham, MA) and 5 × 10^3^ cells were seeded in the upper layer of 35-mm culture dishes. The final concentration of the agar system was 0.6% for the bottom layer and 0.35% for the cell suspension layer. Numbers of colonies were assessed after 2–3 weeks of culture in a humidified incubator at 37°C with 5% CO_2_. Colonies were dyed with 1mL 0.005% crystal violet (Solarbio, Beijing, China) for 1 h at 37°C. Images of colonies were obtained with a GelDoc Imagining instrument (BioRad, Hercules, CA), and colonies were counted under a microscope. In 10 randomly selected fields of view, colonies contained more than 50 cells were counted. All experiments were performed in triplicate.

### Cell invasion assay

HepG2 cells (1 × 10^4^ cells) were suspended in 0.2 mL DMEM medium with 5% FBS and treated with 20 μg/mL of GE11-VLPs-MEG3 and GE11-VLPs-NC for 48 h. Then the cell suspension was seeded into two wells of the upper transwell chamber (8-mm pore size, Corning Corp., NY); one was pre-coated with 100μl BD Matrigel Basement Membrane Matrix (BD Biosciences, Franklin Lakes, NJ), the other was not. In the lower chamber, 0.75 mL DMEM medium with 20% FBS was added. After incubating for 24 h in a humidified incubator at 37°C with 5% CO_2_, chambers were disassembled and the membranes were stained with a Diff-Quik Staining Kit (ZKKA Biotechnology, Beijing, China) following the manufacturer's instructions. The number of cells penetrating across the membrane was counted under a microscope in ten random 400× visual fields and compared with the respective control groups. All experiments were performed in triplicate.

### Cell cycle and cell apoptosis

HepG2 cells for cell cycle analysis were harvested 48 h after treatment with 20 μg/mL of GE11-VLPs-MEG3 and GE11-VLPs-NC. Following staining with propidium iodide (PI, Beyotime Biotechnology, Beijing, China) performed according to the manufacturer's instructions, the cells were analyzed using flow cytometry (BD FACSCalibur, Heidelberg, Germany). The relative ratio of cells in the G0/G1, S, or G2/M phases was counted and compared with the respective control groups.

HepG2 cells for cell apoptosis analysis were harvested by trypsin digestion, washes twice by PBS (pH 7.4) and double-stained with Annexin V-FITC and PI (Dojindo, Kumamoto, Japan) according to the instructions after treatment with 20 μg/mL of GE11-VLPs-MEG3 and GE11-VLPs-NC for 48 h and analyzed using a flow cytometer (BD FACSCalibur, Heidelberg, Germany). Cells were classified as viable, dead, early apoptotic, or apoptotic. The percentage of early and late apoptotic cells was counted and compared between cells receiving different treatments. All the experiments were performed in triplicate.

### Western blot assay

Cells seeded in 6-well plates were lysed using 500μl the mammalian protein extraction reagent RIPA (Beyotime, Beijing, China), together with 1μl protease inhibitor cocktail (Roche, Pleasanton, CA). After centrifugation at 4°C for 30 min at 18,000 *g*, the supernatant was separated and stored on ice. 40μg protein lysates were separated by 12% SDS-PAGE, transferred to 0.22-μm nitrocellulose membranes (Millipore, Boston, MA). The membranes were blocked with 5% fat-free milk for 3 h at room temperature, followed by incubation with primary antibodies against p53 and MDM2 (Santa Cruz, Dallas, TX) at 4°C overnight. An anti-GAPDH antibody (CWBiotech, Beijing, China) was used as a control. Then the membranes were washed five times and incubated with HRP-conjugated secondary antibody (CWBiotech, Beijing, China) for 2 h at room temperature. Signals were visualized using the ECL chromogenic substrate (Thermo scientific, Rockford, IL) and quantified by densitometry using the Quantity One software (Bio-Rad, Berkeley, CA).

### Tumor formation and VLP therapy in a nude mouse model

Three to four-week-old BALB/c nude female mice (SPF/VAF) were purchased from Vital River (Beijing, China) and were kept under SPF isolation conditions and fed a standard diet at the Institute of Laboratory Animal Science (Beijing, China). All animal experiments were approved by the Ethics Committee of the National Center for Clinical Laboratories. HepG2 cells were harvested from T175 cell culture flasks (Corning, NY), washed twice with PBS, and resuspended at a concentration of 1 × 10^8^ cells/mL with BD Matrigel Basement Membrane Matrix (BD, Franklin Lakes, NJ). A volume of 0.1 mL of suspended cells was subcutaneously injected into a single side of the posterior flank of each mouse. Tumor growth was examined every three days, until the tumors were visible to the naked eye and tumor volumes reached approximately 0.5 cm^3^. Tumor volumes were calculated using the equation V = 0.5 × a × b^2^ (V, volume; a, longitudinal diameter; b, latitudinal diameter).

Tumor-bearing mice were randomly divided into three groups: one for therapy with GE11-VLPs-MEG3 (100 ng per administration), one with GE11-VLPs-NC (100 ng per administration) via tail vein administration every three days. In order to further confirm that GE11-VLPs-NC didn’t have the ability of tumor inhibition, we also added a PBS treated group (equal volumes of VLPs). Tumor growth was examined every three days. After 3 weeks’ therapy, mice were euthanized. The tumor tissues were separated and weighed and the subcutaneous growth of each tumor was examined.

### Hematoxylin-eosin (H&E) staining and immunohistochemistry

Tumor tissues isolated from the tumor-bearing mice treated for 3 weeks were fixed 24 h in 4% paraformaldehyde, embedded in paraffin and then sectioned at 5μm. The sections mounted on the glass slides were deparaffinized and rehydrated, then stained with hematoxylin and eosin following the standard protocol. Immunodetection was performed with anti-Ki67 antibody (Abcam, Cambridge, UK) followed by incubation with HRP-conjugated secondary antibodies (ZSGB-Bio, Beijing, China) Images were captured in ten random 200× visual fields.

### Statistical analysis

All experiments were independently repeated in triplicate. Data are expressed as mean ± SD. Differences between two independent groups were analyzed with the Student's *t*-test. All statistical analyses were performed using SPSS version 19.0 and presented using the GraphPad Prism 6 software. The results were considered to be statistically significant at P < 0.05.

## References

[1] Torre LA, Bray F, Siegel RL, Ferlay J, Lortet-Tieulent J, Jemal A (2015). Global cancer statistics, 2012. CA Cancer J Clin.

[2] Maluccio M, Covey A (2012). Recent progress in understanding, diagnosing, and treating hepatocellular carcinoma. CA Cancer J Clin.

[3] Rudalska R, Dauch D, Longerich T, McJunkin K, Wuestefeld T, Kang TW, Hohmeyer A, Pesic M, Leibold J, von Thun A, Schirmacher P, Zuber J, Weiss KH (2014). In vivo RNAi screening identifies a mechanism of sorafenib resistance in liver cancer. Nat Med.

[4] Ling H, Fabbri M, Calin GA (2013). MicroRNAs and other non-coding RNAs as targets for anticancer drug development. Nat Rev Drug Discov.

[5] He Y, Meng XM, Huang C, Wu BM, Zhang L, Lv XW, Li J (2014). Long noncoding RNAs: Novel insights into hepatocelluar carcinoma. Cancer Lett.

[6] Fu WM, Lu YF, Hu BG, Liang WC, Zhu X, Yang HD, Li G, Zhang JF (2015). Long noncoding RNA hotair mediated angiogenesis in nasopharyngeal carcinoma by direct and indirect signaling pathways. Oncotarget.

[7] Lu Z, Xiao Z, Liu F, Cui M, Li W, Yang Z, Li J, Ye L, Zhang X (2016). Long non-coding RNA HULC promotes tumor angiogenesis in liver cancer by up-regulating sphingosine kinase 1 (SPHK1). Oncotarget.

[8] Matouk IJ, Halle D, Raveh E, Gilon M, Sorin V, Hochberg A (2015). The role of the oncofetal H19 lncRNA in tumor metastasis: orchestrating the EMT-MET decision. Oncotarget.

[9] Taniue K, Kurimoto A, Sugimasa H, Nasu E, Takeda Y, Iwasaki K, Nagashima T, Okada-Hatakeyama M, Oyama M, Kozuka-Hata H, Hiyoshi M, Kitayama J, Negishi L (2016). Long noncoding RNA UPAT promotes colon tumorigenesis by inhibiting degradation of UHRF1. Proc Natl Acad Sci U S A.

[10] Zhang X, Zhou Y, Mehta KR, Danila DC, Scolavino S, Johnson SR, Klibanski A (2003). A pituitary-derived MEG3 isoform functions as a growth suppressor in tumor cells. J Clin Endocrinol Metab.

[11] Zhang X, Gejman R, Mahta A, Zhong Y, Rice KA, Zhou Y, Cheunsuchon P, Louis DN, Klibanski A (2010). Maternally expressed gene 3, an imprinted noncoding RNA gene, is associated with meningioma pathogenesis and progression. Cancer Res.

[12] Lu KH, Li W, Liu XH, Sun M, Zhang ML, Wu WQ, Xie WP, Hou YY (2013). Long non-coding RNA MEG3 inhibits NSCLC cells proliferation and induces apoptosis by affecting p53 expression. BMC Cancer.

[13] He Y, Wu YT, Huang C, Meng XM, Ma TT, Wu BM, Xu FY, Zhang L, Lv XW, Li J (2014). Inhibitory effects of long noncoding RNA MEG3 on hepatic stellate cells activation and liver fibrogenesis. Biochim Biophys Acta.

[14] Ying L, Huang Y, Chen H, Wang Y, Xia L, Chen Y, Liu Y, Qiu F (2013). Downregulated MEG3 activates autophagy and increases cell proliferation in bladder cancer. Mol Biosyst.

[15] Wang P, Ren Z, Sun P (2012). Overexpression of the long non-coding RNA MEG3 impairs in vitro glioma cell proliferation. J Cell Biochem.

[16] Jia LF, Wei SB, Gan YH, Guo Y, Gong K, Mitchelson K, Cheng J, Yu GY (2014). Expression, regulation and roles of miR-26a and MEG3 in tongue squamous cell carcinoma. Int J Cancer.

[17] Yin DD, Liu ZJ, Zhang E, Kong R, Zhang ZH, Guo RH (2015). Decreased expression of long noncoding RNA MEG3 affects cell proliferation and predicts a poor prognosis in patients with colorectal cancer. Tumour Biol.

[18] Braconi C, Kogure T, Valeri N, Huang N, Nuovo G, Costinean S, Negrini M, Miotto E, Croce CM, Patel T (2011). microRNA-29 can regulate expression of the long non-coding RNA gene MEG3 in hepatocellular cancer. Oncogene.

[19] Zhuo H, Tang J, Lin Z, Jiang R, Zhang X, Ji J, Wang P, Sun B (2015). The aberrant expression of MEG3 regulated by UHRF1 predicts the prognosis of hepatocellular carcinoma. Mol Carcinog.

[20] Benetatos L, Vartholomatos G, Hatzimichael E (2011). MEG3 imprinted gene contribution in tumorigenesis. Int J Cancer.

[21] Zhan S, Li J, Xu R, Wang L, Zhang K, Zhang R (2009). Armored long RNA controls or standards for branched DNA assay for detection of human immunodeficiency virus type 1. J Clin Microbiol.

[22] Pan Y, Jia T, Zhang Y, Zhang K, Zhang R, Li J, Wang L (2012). MS2 VLP-based delivery of microRNA-146a inhibits autoantibody production in lupus-prone mice. Int J Nanomedicine.

[23] Sun Y, Jia T, Sun Y, Han Y, Wang L, Zhang R, Zhang K, Lin G, Xie J, Li J (2013). External quality assessment for Avian Influenza A (H7N9) Virus detection using armored RNA. J Clin Microbiol.

[24] Wang G, Sun Y, Zhang K, Jia T, Hao M, Zhang D, Chang L, Zhang L, Zhang R, Lin G, Peng R, Li J (2015). External Quality Assessment of Molecular Detection of Ebola Virus in China. PLoS One.

[25] Wei B, Wei Y, Zhang K, Yang C, Wang J, Xu R, Zhan S, Lin G, Wang W, Liu M, Wang L, Zhang R, Li J (2008). Construction of armored RNA containing long-size chimeric RNA by increasing the number and affinity of the pac site in exogenous rna and sequence coding coat protein of the MS2 bacteriophage. Intervirology.

[26] Zhang D, Sun Y, Jia T, Zhang L, Wang G, Zhang R, Zhang K, Lin G, Xie J, Wang L, Li J (2015). External Quality Assessment for the Detection of Measles Virus by Reverse Transcription-PCR Using Armored RNA. PLoS One.

[27] Li J, Sun Y, Jia T, Zhang R, Zhang K, Wang L (2014). Messenger RNA vaccine based on recombinant MS2 virus-like particles against prostate cancer. Int J Cancer.

[28] Pan Y, Zhang Y, Jia T, Zhang K, Li J, Wang L (2012). Development of a microRNA delivery system based on bacteriophage MS2 virus-like particles. FEBS J.

[29] Caldeira JC, Peabody DS (2011). Thermal stability of RNA phage virus-like particles displaying foreign peptides. J Nanobiotechnology.

[30] Lima SM, Vaz AC, Souza TL, Peabody DS, Silva JL, Oliveira AC (2006). Dissecting the role of protein-protein and protein-nucleic acid interactions in MS2 bacteriophage stability. FEBS J.

[31] Li Z, Zhao R, Wu X, Sun Y, Yao M, Li J, Xu Y, Gu J (2005). Identification and characterization of a novel peptide ligand of epidermal growth factor receptor for targeted delivery of therapeutics. FASEB J.

[32] Mickler FM, Mockl L, Ruthardt N, Ogris M, Wagner E, Brauchle C (2012). Tuning nanoparticle uptake: live-cell imaging reveals two distinct endocytosis mechanisms mediated by natural and artificial EGFR targeting ligand. Nano Lett.

[33] Schafer A, Pahnke A, Schaffert D, van Weerden WM, de Ridder CM, Rodl W, Vetter A, Spitzweg C, Kraaij R, Wagner E, Ogris M (2011). Disconnecting the yin and yang relation of epidermal growth factor receptor (EGFR)-mediated delivery: a fully synthetic, EGFR-targeted gene transfer system avoiding receptor activation. Hum Gene Ther.

[34] Ohno S, Takanashi M, Sudo K, Ueda S, Ishikawa A, Matsuyama N, Fujita K, Mizutani T, Ohgi T, Ochiya T, Gotoh N, Kuroda M (2013). Systemically injected exosomes targeted to EGFR deliver antitumor microRNA to breast cancer cells. Mol Ther.

[35] Ahsan A, Ramanand SG, Bergin IL, Zhao L, Whitehead CE, Rehemtulla A, Ray D, Pratt WB, Lawrence TS, Nyati MK (2014). Efficacy of an EGFR-specific peptide against EGFR-dependent cancer cell lines and tumor xenografts. Neoplasia.

[36] Tang H, Chen X, Rui M, Sun W, Chen J, Peng J, Xu Y (2014). Effects of surface displayed targeting ligand GE11 on liposome distribution and extravasation in tumor. Mol Pharm.

[37] Su YH, Ng KF, Yu MC, Wu TJ, Yeh TS, Lee WC, Lin YS, Hsieh TH, Lin CY, Yeh CT, Chen TC (2015). Impact of epidermal growth factor receptor protein and gene alteration on Taiwanese hepatocellular carcinomas. J Gastroenterol Hepatol.

[38] Buckley AF, Burgart LJ, Sahai V, Kakar S (2008). Epidermal growth factor receptor expression and gene copy number in conventional hepatocellular carcinoma. Am J Clin Pathol.

[39] Hussain KM, Leong KL, Ng MM, Chu JJ (2011). The essential role of clathrin-mediated endocytosis in the infectious entry of human enterovirus 71. J Biol Chem.

[40] Mickler FM, Vachutinsky Y, Oba M, Miyata K, Nishiyama N, Kataoka K, Brauchle C, Ruthardt N (2011). Effect of integrin targeting and PEG shielding on polyplex micelle internalization studied by live-cell imaging. J Control Release.

[41] Liu J, Shapiro JI (2003). Endocytosis and signal transduction: basic science update. Biol Res Nurs.

[42] Wu X, Tai Z, Zhu Q, Fan W, Ding B, Zhang W, Zhang L, Yao C, Wang X, Ding X, Li Q, Li X, Liu G (2014). Study on the prostate cancer-targeting mechanism of aptamer-modified nanoparticles and their potential anticancer effect in vivo. Int J Nanomedicine.

[43] Meng X, Franklin DA, Dong J, Zhang Y (2014). MDM2-p53 pathway in hepatocellular carcinoma. Cancer Res.

[44] Cheng L, Huang FZ, Cheng LF, Zhu YQ, Hu Q, Li L, Wei L, Chen DW (2014). GE11-modified liposomes for non-small cell lung cancer targeting: preparation, ex vitro and in vivo evaluation. Int J Nanomedicine.

[45] Meng S, Zhan S, Li J (2009). Nuclease-resistant double-stranded DNA controls or standards for hepatitis B virus nucleic acid amplification assays. Virol J.

[46] Song L, Sun S, Li B, Pan Y, Li W, Zhang K, Li J (2011). External quality assessment for enterovirus 71 and coxsackievirus A16 detection by reverse transcription-PCR using armored RNA as a virus surrogate. J Clin Microbiol.

[47] Yao Y, Jia T, Pan Y, Gou H, Li Y, Sun Y, Zhang R, Zhang K, Lin G, Xie J, Li J, Wang L (2015). Using a novel microRNA delivery system to inhibit osteoclastogenesis. Int J Mol Sci.

[48] Zhou Y, Zhong Y, Wang Y, Zhang X, Batista DL, Gejman R, Ansell PJ, Zhao J, Weng C, Klibanski A (2007). Activation of p53 by MEG3 non-coding RNA. J Biol Chem.

[49] Zhang A, Xu M, Mo YY (2014). Role of the lncRNA-p53 regulatory network in cancer. J Mol Cell Biol.

[50] Lanz RB, McKenna NJ, Onate SA, Albrecht U, Wong J, Tsai SY, Tsai MJ, O'Malley BW (1999). A steroid receptor coactivator, SRA, functions as an RNA and is present in an SRC-1 complex. Cell.

[51] Li PX, Wong J, Ayed A, Ngo D, Brade AM, Arrowsmith C, Austin RC, Klamut HJ (2000). Placental transforming growth factor-beta is a downstream mediator of the growth arrest and apoptotic response of tumor cells to DNA damage and p53 overexpression. J Biol Chem.

[52] Baek SJ, Kim KS, Nixon JB, Wilson LC, Eling TE (2001). Cyclooxygenase inhibitors regulate the expression of a TGF-beta superfamily member that has proapoptotic and antitumorigenic activities. Mol Pharmacol.

[53] Zhang X, Rice K, Wang Y, Chen W, Zhong Y, Nakayama Y, Zhou Y, Klibanski A (2010). Maternally expressed gene 3 (MEG3) noncoding ribonucleic acid: isoform structure, expression, and functions. Endocrinology.

[54] Zhou Y, Zhang X, Klibanski A (2012). MEG3 noncoding RNA: a tumor suppressor. J Mol Endocrinol.

[55] Lowary PT, Uhlenbeck OC (1987). An RNA mutation that increases the affinity of an RNA-protein interaction. Nucleic Acids Res.

[56] Parrott AM, Lago H, Adams CJ, Ashcroft AE, Stonehouse NJ, Stockley PG (2000). RNA aptamers for the MS2 bacteriophage coat protein and the wild-type RNA operator have similar solution behaviour. Nucleic Acids Res.

[57] Horn WT, Convery MA, Stonehouse NJ, Adams CJ, Liljas L, Phillips SE, Stockley PG (2004). The crystal structure of a high affinity RNA stem-loop complexed with the bacteriophage MS2 capsid: further challenges in the modeling of ligand-RNA interactions. RNA.

